# Supplementary material for: Bone Mineral Density and Trabecular Bone Score Changes throughout Menopause in Women with HIV
Source: Viruses. 2023 Dec 1;15(12):2375. doi: 10.3390/v15122375 (PMC10747369; doi:10.3390/v15122375)
Supplement: Supplementary file 1 [file viruses-15-02375-s001.zip › viruses-2423227-supplementary.pdf]

Supplementary Table S1 depicts STRAW periods according to endocrine and menstrual cycle criteria. The main criteria for menopause were the absence of menstrual bleeding for more than 1 year or history of total abdominal hysterectomy. Supportive criteria used in this study included biomarkers of ovary insufficiency.

Final menstrual period

**Stage**			**+1a** **+1b**	**+1c** **+2**
**TERMS**	*Reproductive period*	*Menopause transition period*	*Early menopause period*	*Late menopause period*
**MENSTRUAL CYCLE**	Regular	Variable length with intervals of amenorrhea of >= 60 days	Amenorrhea	Amenorrhea
**ENDOCRINE CRITERIA**			Biomarkers of ovary insufficiency: LH>25ng/mL; FSH>25 ng/mL; E2<30 ng/mL	

Supplementary Table S2. Trends of BMD across menopause periods according to different risk factors.

Supplementary Table S2A. Estimations of BMD trends in WWH according to use of bisphosphonates.

	Estimate	p
No: (Early menopause - Menopause transition) vs (Menopause transition - Reproductive)	-0.046	0.003
No: (Late menopause - Early menopause) vs (Menopause transition - Reproductive)	-0.054	<0.001
No: (Late menopause - Early menopause) vs (Early menopause - Menopause transition)	-0.008	0.511
Yes: (Early menopause - Menopause transition) vs (Menopause transition - Reproductive)	-0.074	0.077
Yes: (Late menopause - Early menopause) vs (Menopause transition - Reproductive)	-0.054	0.041
Yes: (Late menopause - Early menopause) vs (Menopause transition - Reproductive)	0.020	0.433
(Menopause transition - Reproductive): No vs Yes	-0.068	0.003
(Early menopause - Menopause transition): No vs Yes	-0.039	0.059
(Late menopause - Early menopause): No vs Yes	-0.068	<0.001
Reproductive: No vs Yes	0.138	<0.001
Menopause transition: No vs Yes	0.070	0.001
Early menopause: No vs Yes	0.030	0.001
Late menopause: No vs Yes	-0.038	0.013

Supplementary Table S2B. Estimations of BMD trends in WWH according to kidney function [eGFR < 60 (yes) vs ≥ 60 ml/min/1.73m^2 (no)].

	Estimate	p
No: (Early menopause - Menopause transition) vs (Menopause transition - Reproductive)	-0.060	0.004
No: (Late menopause - Early menopause) vs (Menopause transition - Reproductive)	-0.064	<0.001
No: (Late menopause - Early menopause) vs (Early menopause - Menopause transition)	-0.004	0.783
Yes: (Early menopause - Menopause transition) vs (Menopause transition - Reproductive)	-0.060	0.087
Yes: (Late menopause - Early menopause) vs (Menopause transition - Reproductive)	-0.044	0.051
Yes: (Late menopause - Early menopause) vs (Menopause transition - Reproductive)	0.016	0.513
(Menopause transition - Reproductive): No vs Yes	-0.014	0.368
(Early menopause - Menopause transition): No vs Yes	-0.015	0.381
(Late menopause - Early menopause): No vs Yes	-0.034	0.016
Reproductive: No vs Yes	0.016	0.253
Menopause transition: No vs Yes	0.001	0.930
Early menopause: No vs Yes	-0.013	0.157
Late menopause: No vs Yes	-0.047	0.002

Supplementary Table S2C. Estimations of BMD trends in WWH according to BMI [BMI ≥ 25 (yes) vs. <25 kg/m^2 (no)].

	Estimate	p
No: (Early menopause - Menopause transition) vs (Menopause transition - Reproductive)	-0.063	0.010
No: (Late menopause - Early menopause) vs (Menopause transition - Reproductive)	-0.073	<0.001
No: (Late menopause - Early menopause) vs (Early menopause - Menopause transition)	-0.009	0.580
Yes: (Early menopause - Menopause transition) vs (Menopause transition - Reproductive)	-0.057	0.041
Yes: (Late menopause - Early menopause) vs (Menopause transition - Reproductive)	-0.036	0.058
Yes: (Late menopause - Early menopause) vs (Menopause transition - Reproductive)	0.021	0.266
(Menopause transition - Reproductive): No vs Yes	-0.001	0.914
(Early menopause - Menopause transition): No vs Yes	-0.008	0.458
(Late menopause - Early menopause): No vs Yes	-0.038	0.005
Reproductive: No vs Yes	-0.022	0.024
Menopause transition: No vs Yes	-0.023	0.037
Early menopause: No vs Yes	-0.030	<0.001
Late menopause: No vs Yes	-0.069	<0.001

Supplementary Table S2D. Estimations of BMD trends in WWH according to use of TDF.

	Estimate	p
No: (Early menopause - Menopause transition) vs (Menopause transition - Reproductive)	-0.060	0.014
No: (Late menopause - Early menopause) vs (Menopause transition - Reproductive)	-0.054	0.001
No: (Late menopause - Early menopause) vs (Early menopause - Menopause transition)	0.006	0.707
Yes: (Early menopause - Menopause transition) vs (Menopause transition - Reproductive)	-0.060	0.014
Yes: (Late menopause - Early menopause) vs (Menopause transition - Reproductive)	-0.054	0.001
Yes: (Late menopause - Early menopause) vs (Menopause transition - Reproductive)	0.006	0.707
Menopause transition: No vs Yes	0.025	<0.001
Early menopause: No vs Yes	0.025	<0.001
Late menopause: No vs Yes	0.025	<0.001

Supplementary Table S2E. Estimations of BMD trends in WWH according to use of PI.

	Estimate	p
No: (Early menopause - Menopause transition) vs (Menopause transition - Reproductive)	-0.087	0.001
No: (Late menopause - Early menopause) vs (Menopause transition - Reproductive)	-0.072	<0.001
No: (Late menopause - Early menopause) vs (Early menopause - Menopause transition)	0.015	0.435
Yes: (Early menopause - Menopause transition) vs (Menopause transition - Reproductive)	-0.033	0.187
Yes: (Late menopause - Early menopause) vs (Menopause transition - Reproductive)	-0.036	0.026
Yes: (Late menopause - Early menopause) vs (Menopause transition - Reproductive)	-0.003	0.863
(Menopause transition - Reproductive): No vs Yes	0.034	0.001
(Early menopause - Menopause transition): No vs Yes	-0.020	0.035
(Late menopause - Early menopause): No vs Yes	-0.002	0.856
Reproductive: No vs Yes	0.011	0.227
Menopause transition: No vs Yes	0.045	<0.001
Early menopause: No vs Yes	0.025	0.001
Late menopause: No vs Yes	0.023	0.109

Supplementary Table S2F. Estimations of BMD trends in WWH according to current CD4 cell count [current CD4 cell count ≥ 500 (yes) vs. <500 c/microL (no)].

	Estimate	p
No: (Early menopause - Menopause transition) vs (Menopause transition - Reproductive)	-0.060	0.014
No: (Late menopause - Early menopause) vs (Menopause transition - Reproductive)	-0.054	0.001
No: (Late menopause - Early menopause) vs (Early menopause - Menopause transition)	0.006	0.707
Yes: (Early menopause - Menopause transition) vs (Menopause transition - Reproductive)	-0.060	0.014
Yes: (Late menopause - Early menopause) vs (Menopause transition - Reproductive)	-0.054	0.001
Yes: (Late menopause - Early menopause) vs (Menopause transition - Reproductive)	0.006	0.707
Reproductive: No vs Yes	0.017	0.043
Menopause transition: No vs Yes	0.017	0.043
Early menopause: No vs Yes	0.017	0.043
Late menopause: No vs Yes	0.017	0.043

Supplementary Table S3. Trends of TBS across menopause periods according to different risk factors.

Supplementary Table S3A. Estimations of TBS trends in WWH according to use of bisphosphonates.

	**Estimate**	**p**
No: (Menopause - Menopause transition) vs (Menopause transition - Reproductive)	-0.052	0.012
Yes: (Menopause - Menopause transition) vs (Menopause transition - Reproductive)	-0.052	0.012
Reproductive: No vs Yes	0.073	0.002
Menopause transition: No vs Yes	0.073	0.002
Menopause: No vs Yes	0.073	0.002

Supplementary Table S3B. Estimations of TBS trends in WWH according to smoking status.

	**Estimate**	**p**
No: (Menopause - Menopause transition) vs (Menopause transition - Reproductive)	-0.052	0.012
Yes: (Menopause - Menopause transition) vs (Menopause transition - Reproductive)	-0.052	0.012
Reproductive: No vs Yes	0.034	0.041
Menopause transition: No vs Yes	0.034	0.041
Menopause: No vs Yes	0.034	0.041

Supplementary Table S3C. Estimations of TBS trends in WWH according to BMI [BMI ≥ 25 (yes) vs. <25 kg/m^2 (no)].

	**Estimate**	**p**
No: (Menopause - Menopause transition) vs (Menopause transition - Reproductive)	-0.083	<0.001
Yes: (Menopause - Menopause transition) vs (Menopause transition - Reproductive)	-0.021	0.546
(Menopause transition - Reproductive): No vs Yes	0.014	0.604
(Menopause - Menopause transition): No vs Yes	-0.049	0.015
Reproductive: No vs Yes	0.022	0.380
Menopause transition: No vs Yes	0.035	0.130
Menopause: No vs Yes	-0.014	0.462

Supplementary Table S3D. Estimations of TBS trends in WWH according to use of PI.

	**Estimate**	**p**
No: (Menopause - Menopause transition) vs (Menopause transition - Reproductive)	-0.099	0.001
Yes: (Menopause - Menopause transition) vs (Menopause transition - Reproductive)	-0.006	0.813
(Menopause transition - Reproductive): No vs Yes	0.035	0.150
(Menopause - Menopause transition): No vs Yes	-0.058	0.002
Reproductive: No vs Yes	0.007	0.753
Menopause transition: No vs Yes	0.041	0.037
Menopause: No vs Yes	-0.017	0.316

Supplementary Figure S1. depicts trajectories of BMD Z-score across menopause. Panel A presents the slope coefficient of BMD Z-score change in the 4 STRAW+10 intervals. Panel B describes the average values of BMD Z-score at each transition period. The comparison derived from STRAW+10 periods: (i) reproductive, (ii) menopause transition, (iii) early menopause, and (iv) late menopause.

A

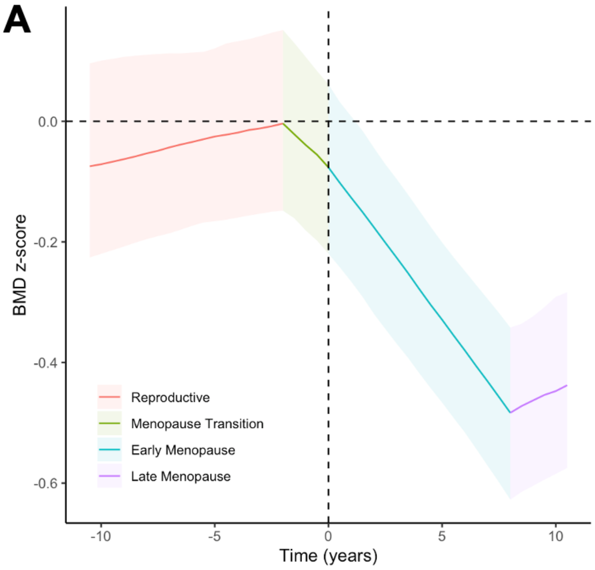

Predictors	Estimates	p
T1	0.01	0.202
T2	-0.04	0.071
T3	-0.05	<0.001
T4	0.02	0.128

	Coefficients	p
T2 - T1	-0.0454579	0.06
T3 - T1	-0.0594053	<0.001
T4 - T1	0.0116170	0.42
T3 - T2	-0.0139474	0.56
T4 - T2	0.0570750	0.01
T4 - T3	0.0710223	<0.001

B

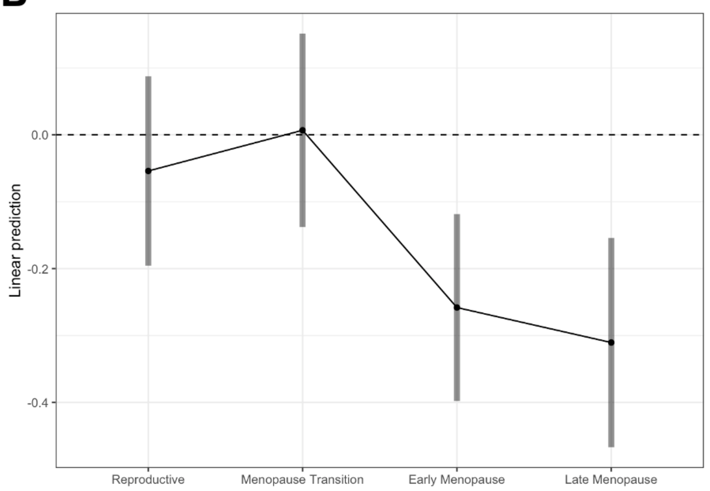

	Estimates	p
Reproductive: HIV cohort vs reference population	-0.054	0.453
Menopause Transition: HIV cohort vs reference population	0.007	0.925
Early Menopause: HIV cohort vs reference population	-0.258	<0.001
Late Menopause: HIV cohort vs reference population	-0.311	<0.001
